# Society Meetings

**Published:** 1898-03

**Authors:** 


### Society Meetings.

---

THE Buffalo Academy of Medicine held meetings during the month of February, 1898, as follows :

*Section on Surgery.*—Tuesday evening, February 1st, program : The technique of hysterectomy, both vaginal and abdominal, by Dr. Dudley P. Allen, professor of surgery and clinical surgery, Western Reserve University, medical department, Cleveland, O. Exhibition of a case of penetrating gunshot wound of the head, by Dr. Eugene A. Smith.

*Section on Medicine.*—Tuesday evening, February 8th, program : Clinical examination of blood, by Dr. Helene Kuhlman. Sputum, by Dr. Nelson Russell.

*Section on Pathology.*—Tuesday evening, February 15th, program : Toxic degeneration of the nervous system, by Prof. Ira Van Gieson, director pathological institute, New York state commission in lunacy ; discussion by Drs. Crego, Hurd and Krauss.

*Section on Obstetrics and Gynecology.*—Tuesday evening, February 22d, program : Etiology, pathology, diagnosis and treatment of myofibroma of the uterus, by Dr. Henry D. Ingraham.

---

THE American Academy of Medicine will hold its twenty-third annual meeting at Denver, Colorado, on Saturday, June 4th, and Monday, June 6, 1898, in the Brown Palace Hotel. It is planned to hold three sessions on Saturday beginning at 10 A. M., at 3.00 and at 8.00 P. M. Another session will be held on Monday, at 10 A. M., at which time it is hoped all the business can be completed, affording the fellows an opportunity to attend the meetings either of the Association of American Medical Colleges or the National Confederation of State Medical Examining and Licensing Boards, which meet on this day. The reunion session will be held Monday evening. The following papers have been promised :

Dr. L. Duncan Bulkley, of New York, the president's address ; Dr. Charles Denison, of Denver, The advantage of physical education as a prevention of disease ; Dr. Thomas C. Ely, of Philadelphia, The importance of training the special senses in the education of children ;

Dr. J. Edgar Fretz, of Easton, Pa., Some criticisms on the questions of the medical examining boards by a recent graduate ; Dr. G. G. Groff, of Bucknell University, Lewisburg, Pa., The necessity of medical supervision in school-life ; Dr. Bayard Holmes, of Chicago, Growth and strength and its relation to education and medicine ; Dr. Henry M. Hurd, of Baltimore, How much to educate the growing brain ; Dr. Woods Hutchinson, of Buffalo, The muscular basis of education ; Dr. Edward Jackson, of Philadelphia, The care of the eyes during school-life ; Dr. Elmer Lee, of New York, The interdependence of healthy bodies and healthy brains ; Dr. J. C. Lichty, Clifton Springs, N. Y., The modern sanitarium and its relation to the general medical profession ; Dr. Charles McIntire, of Easton, Pa., Snags in the course of the medical examining boards ; Dr. Rupert Norton, of Washington, D. C., The child's brain as illustrated by recent neurologic studies ; Dr. F. T. Rogers, of Providence, R. I., The ethical advertiser ; Dr. J. T. Searcy, of Tuscaloosa, Ala., How education fails—physiologically considered ; Dr. Charles G. Stockton, of Buffalo, The kindergarten ; Dr. James L. Taylor, of Wheelersburg, O., The amount of work a growing brain ought to undertake ; Dr. Casey A. Wood, of Chicago, Kindergarten and primary grade work in the public schools and its influence upon the eyesight.

Fellows desiring to contribute are requested to send their titles to the secretary, Dr. Charles McIntire, Easton, Pa.

---

The Medical Society of the State of New York at its last meeting elected the following-named officers and committees to serve for the ensuing year : President, John O. Roe, 28 North Clinton street, Rochester ; vice-president, E. F. Brush, Mount Vernon ; secretary, Frederic C. Curtis, 17 Washington avenue, Albany ; treasurer, Charles H. Porter, 103 Lancaster street, Albany. Committee of arrangements, Samuel B. Ward, 281 State street, Albany ; Wm. J. Nellis, Albany ; Reynold W. Wilcox, New York. Committee on by-laws, H. D. Wey, Elmira ; Nathan Jacobson, Syracuse ; F. C. Curtis, Albany. Committee on hygiene, Henry R. Hopkins, 433 Franklin street, Buffalo ; E. H. Bartley, Brooklyn ; C. E. Bruce, New York ; J. M. Mosher, Albany ; O. W. Peck, Oneonta ; H. F. Hart, Shrub Oak ; George Seymour, Utica. Committee on legislation, Frank Van Fleet, 116 East 82d street, New York ; Arthur G. Root, Albany ; Ernest Wende, Buffalo. Committee on ethics, Evarts M. Morrell, City Island ; George McNaughton, Brooklyn ; Louis A. Weigel, Rochester. Committee on prize essays, J. M. Van Cott, Brooklyn ; Andrew MacFarlane, Albany ; William S. Cheesman, Auburn. Committee of publication, F. C. Curtis, Albany ; Daniel Lewis, New York ; William Warren Potter, Buffalo ; Charles H. Porter, Albany.

The next annual meeting of the society will be held at Albany, January 24 to 26, 1899.

---

THE national confederation of state medical examining and licensing boards will hold its eighth annual meeting, Monday, June 6, 1898, at the Brown Palace Hotel, Denver, Colorado, at 10 o'clock A. M. The following are the officers and committees for the current year : President, Dr. William Warren Potter, 284 Franklin street, Buffalo, N. Y.; vice-presidents, Dr. E. L. B. Godfrey, Camden, N. J., and Dr. Wm. Bailey, Louisville, Ky.; secretary and treasurer, Dr. A. Walter Suiter, Herkimer, N. Y. Executive council, Dr. William S. Foster, chairman, Pittsburg, Pa.; Dr. Joseph M. Mathews, Louisville, Ky.; Dr. Hugh M. Taylor, Richmond, Va. ; Dr. W. H. Sanders, Montgomery, Ala., and Dr. Charles K. Cole, Helena, Montana. Committee on minimum standards, Dr. N. R. Coleman, chairman, Columbus, O.; Dr. Gardner T. Swarts, Providence, R. I. ; Dr. T. J. Happel, Trenton, Tenn. ; Dr. Augustus Korndoerfer, Philadelphia, Pa., and Dr. W. R. Tipton, Las Vegas, N. M.

---

The American Medico-Psychological Association will hold its fifty-fourth annual meeting at St. Louis, Mo., May 10–13, 1898. Arrangements have been made by the committee for the accommodation of members at the Southern hotel, where all meetings of the association will be held. Those interested should communicate with the secretary, Dr. C. B. Burr, Flint, Mich.

---

THE Niagara Falls Academy of Medicine held its regular monthly meeting at the office of Dr. Leo-Wolf, Monday evening, February 7, 1898. Dr. J. H. Miller read a paper entitled Empyemia, which was discussed by the members present. Dr. McBlain's paper, read at the January meeting, entitled Chronic and acute ovaritis, was also discussed. After attending to some business matters the meeting then adjourned.

